# Draft Genome Sequence of the Phototropic Cyanobacterium *Rivularia* sp. Strain IAM M-261

**DOI:** 10.1128/MRA.00790-21

**Published:** 2021-09-30

**Authors:** Yuu Hirose, Mitsunori Katayama

**Affiliations:** a Department of Applied Chemistry and Life Science, Toyohashi University of Technology, Toyohashi, Aichi, Japan; b College of Industrial Technology, Nihon University, Narashino, Chiba, Japan; Montana State University

## Abstract

*Rivularia* sp. strain IAM M-261 is a filamentous cyanobacterium with tapering morphology and basal-apical polarity. The apical filament of this cyanobacterium exhibits positive phototropism toward visible light. To elucidate the molecular basis for this phototropism, we determined the draft genome sequence of this strain.

## ANNOUNCEMENT

*Rivularia* sp. strain IAM M-261 (hereafter, M-261) is a filamentous cyanobacterium that was originally isolated from a cement wall in Bangkok, Thailand, deposited under accession number 8248 at the Thailand Institute of Scientific and Technological Research (TISTR) (https://www.tistr.or.th/tistr_culture/cdetail.php?type=a&tno=8248). This strain was deposited at the IAM culture collection in Japan and is now maintained by the culture collection of the National Institute of Environmental Studies (NIES) in Japan. M-261 has a tapered filament with basal-apical polarity and can develop a heterocyst, a specialized cell for nitrogen fixation, in the basal position of the filament (1). M-261 can reorient the growth direction of its apical filaments toward incident light, i.e., phototropism (2, 3). Phototropism is common in many eukaryotes, such as higher plants, mosses, ferns, yellow-green algae, and fungi (4), but rare in prokaryotes, including cyanobacteria. Phylogenetic analysis based on the 16S rRNA gene sequence showed that M-261 belongs to *Calothrix* cluster 1.1, proposed by Rippka et al. (2, 5, 6). However, a whole-genome sequence of M-261 is not available in the public databases, although information is available for the distantly related *Rivularia* sp. strain PCC 7116 (7) and *Calothrix* sp. strain 336/3 (8).

We performed whole-genome sequencing of M-261 using the MiSeq platform (Illumina). M-261 cells were grown on BG11 medium supplemented with 1% agar at 30°C under an irradiance of 30 μmol photons m^−2^ s^−1^ provided by fluorescent lamps. Genomic DNA from M-261 was prepared using the Wizard genomic DNA purification kit (Promega) and further purified using a Genomic-tip 20/G kit (Qiagen). Paired-end libraries with an insert size of ∼800 bp were prepared using the TruSeq DNA PCR-free library prep kit (Illumina). Mate-pair libraries with an insert size of ∼8 kbp were prepared using the Nextera mate pair sample preparation kit (Illumina). Each 300-bp end of the libraries was sequenced on a MiSeq instrument using the MiSeq reagent kit v3 (600 cycles; Illumina). Base calling and demultiplexing of the reads were performed using Real-Time Analysis v1.18.54 and MiSeq Control Software v2.6.21. Correction of sequence errors based on a 17-mer frequency and removal of junction sequences of the mate-pair reads were performed using ShortReadManager (9). In total, 2.13 million paired-end reads (total, 431 Mbp) and 1.60 million mate-pair reads (total, 81.7 Mbp) were assembled using Newbler v2.9 (Roche). In total, 11,292,443 bp of the genome was successfully assembled with 35× coverage of the sequence reads, which consisted of 118 scaffolds (>2 kbp) with an *N*_50_ scaffold size of 806 kbp and contained 985 contigs with an *N*_50_ contig size of 16.3 kbp. A total of 9,175 coding sequences, 63 tRNAs, and 19 CRISPR genes were predicted and annotated using the DFAST pipeline (10). The GC content and coding ratio of the genome were calculated as 38.5% and 77.1%, respectively. The axenicity of the genome was checked using CheckM, with a completeness of 96.15%, contamination of 0.95%, and strain heterogeneity of 16.67% (11). Default parameters were used for all software unless otherwise noted. The draft genome sequence of M-261 will provide new information pertaining to the molecular mechanism by which phototropism of cyanobacteria is regulated.

### Data availability.

The draft genome sequence of M-261 was deposited at the DNA Data Bank of Japan (DDBJ) under accession numbers BPQA01000001 to BPQA01000118. The raw sequence reads used for the assembly were deposited at the DDBJ Sequence Read Archive under accession number DRA012446.
